# Collet-Sicard Syndrome from Thrombosis of the Sigmoid-Jugular Complex: A Case Report and Review of the Literature

**DOI:** 10.1155/2010/203587

**Published:** 2010-07-25

**Authors:** Tom P. B. Handley, Mohammed S. Miah, Samit Majumdar, S. S. Musheer Hussain

**Affiliations:** Department of Otolaryngology-Head & Neck Surgery, Ninewells Hospital & Medical School, Dundee DD1 9SY, UK

## Abstract

*Purpose*. Collet-Sicard syndrome is a very rare condition characterised by unilateral palsy of the IX–XII cranial nerves. It is distinguished from Villaret syndrome by lack of presence of sympathetic involvement. Current literature contains only two cases of Collet-Sicard syndrome due to idiopathic internal jugular vein thrombosis. *Method and Results*. We report the case of Collet-Sicard syndrome in a 30-year-old man who presented with delayed development of XIth nerve dysfunction, due to internal jugular vein-sigmoid sinus thrombosis. A multidisciplinary team approach was employed in the management of this patient. At three-month followup, he had significantly improved swallowing, and repeat computed tomography neck scan showed partial recanalisation of the right internal jugular vein. *Conclusion*. In suspected Collet-Sicard syndrome, a focal primary lesion or metastasis to the temporal bone must be excluded, and sigmoid-jugular complex thrombosis should be considered in the differential diagnosis. Early recognition and treatment may result in significant functional recovery.

## 1. Introduction

Venous thromboembolism has an annual incidence of 1 in a 1000 in developed countries [1]. It most commonly affects the lower extremity veins but can occur in any vein. The veins in the head and neck region appear less susceptible to thrombosis even in the presence of local causes due to being valveless and gravity assists in emptying the veins when in the upright position [1]. Causes include direct and iatrogenic trauma, vascular causes including thrombosis and malignancy [1]. With the advent of antibiotics and their widespread use, there has been a marked fall in otogenic and deep neck space infections [1]. Consequently, thrombosis of internal jugular vein (IJV) is rare. This paper describes a unique case of idiopathic IJV-sigmoid sinus thrombosis resulting in Collet-Sicard syndrome (CSS) with delayed presentation of the XIth cranial nerve (CN) dysfunction.

## 2. Case Report

A 32-year-old man presented with a gradual onset, severe right sided headache, which resolved spontaneously within 24 hours with the subsequent development of dysphagia, dysphonia, and right-sided neck pain. He had a preceding history of upper respiratory tract infection with frontal sinus pain, fevers, and mucopurulent nasal discharge. He had no significant past medical or family history. He denied any illicit drug abuse, was a non-smoker, and consumed moderate alcohol.

On examination, the right neck was tender along the deep cervical chain, but with no lymphadenopathy. His ears, nose, and neck movements were normal. Examination of the oropharynx and larynx revealed dysfunction of the right-sided IXth, Xth, and XIIth CNs, with loss of right-sided gag reflex, paralysis of right soft palate musculature with uvula deviation to the left, a hypotonic right tongue with deviation to the right on protrusion, and a right-sided vocal cord palsy with pooling of saliva in the hypopharynx. The trapezius and sternocleidomastoid (SCM) muscles were preserved with normal strength, indicating the XIth CN was preserved. The rest of neurological examination was normal. Audiometry was also normal.

**Figure 1:**
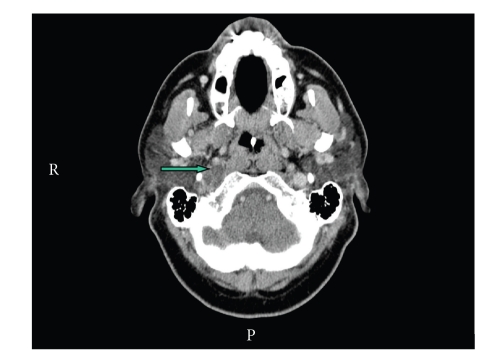
CT neck with contrast showing a low attenuating collection within the right carotid space, which demonstrates peripheral ring enhancement (block arrow). The appearances are suggestive of an abscess. No intracranial abnormality was identified.

**Figure 2:**
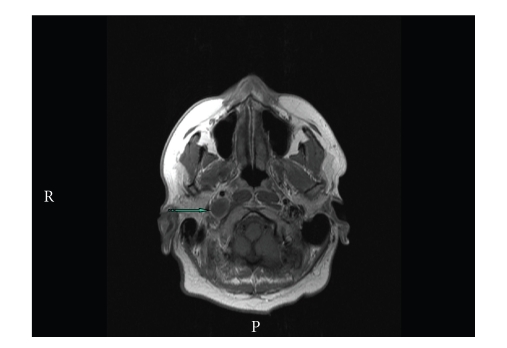
MR neck with contrast demonstrating focal thrombus within the most superior part of the right internal jugular vein and extending through the jugular foramen into the sigmoid sinus (striped arrow). No evidence of deep cerebral venous thrombosis was noted.

Routine blood investigations were normal including white cell count and C-reactive protein. A mild lymphopenia was noted on admission and subsequent testing for HIV proved to be negative. A computed tomography (CT) scan of the neck suggested an abscess in the right carotid space ascending into the jugular foramen (Figure 1). However, magnetic resonance imaging (MRI) confirmed it to be a focal thrombus within the most superior part of the right IJV extending through the jugular foramen into the sigmoid sinus (Figure 2). There was no deep cerebral venous thrombosis. 

Following discussion with a microbiologist, a two-week course of intravenous clindamycin and ceftriaxone were commenced to treat a possible infective cause. Following a fibreoptic endoscopic evaluation of swallowing (FEES), he was placed nil by mouth and nasogastric feeding commenced as silent aspiration was noted. Speech and language therapy were involved in the assessment and rehabilitation. Causes for the thrombosis were sought, in particular neoplastic causes via tumour markers, and a CT scan of the thorax, abdomen, and pelvis proved normal. Investigations for connective tissue and vasculitic causes were negative and tests for an underlying thrombophilia were normal except for a weakly positive lupus anticoagulant. He was subsequently anticoagulated. Following a repeat FEES, a safe swallow was observed and he was discharged on oral diet with outpatient followup.

**Figure 3:**
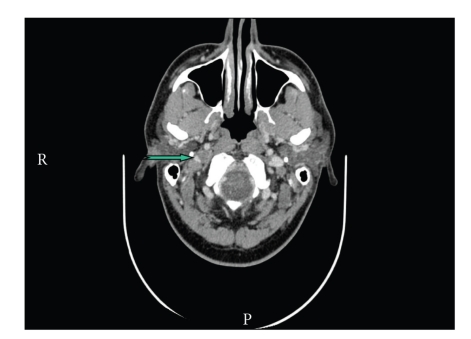
CT neck with contrast at three-month followup showing that a focal filling defect consistent with thrombus is noted in the distal sigmoid sinus on the right side extending into the right internal jugular vein up to the level of C2 vertebra. There is partial recanalisation of the previously thrombosed segment of the right internal jugular vein (notched arrow). Normal appearances of the internal carotid artery and internal jugular vein on the left side are shown.

At six-week followup, he had no resolution of the CN palsies but had also developed CN XI palsy with a reduction in right shoulder strength and atrophy of trapezius and SCM on the right. This confirmed the development of CSS, with the dysfunction of CN IX–XII. At three months followup, he had significantly improved swallowing, and repeat CT neck showed partial recanalisation of right IJV (Figure 3).

## 3. Discussion

Thrombosis of the IJV is an uncommon condition that may lead to fatal complications. The most serious recognised complications are pulmonary emboli with an incidence of 5% [2], and in the presence of infection, septic embolisation. The leading causes of IJV thrombosis are iatrogenic trauma due to jugular venous catheterisation, cardiac pacemaker implantation, repeated venous access by IV drug abusers, and surgery involving the neck [2]. Other causes include malignancy, both primary and metastatic, deep space neck infections, ovarian hyperstimulation in assisted conception therapy [3, 4], and hypercoagulable disorders. Prior studies have observed as much as a 30%–54% incidence of hypercoagulable state in patients with upper extremity deep venous thrombosis (DVT) [5–8].

Headache complicates approximately 80% of cerebral venous sinus thrombosis [9] as in this case with sigmoid sinus involvement. Thrombosis of the sigmoid-jugular complex is frequently asymptomatic because the venous drainage system of the brain has sufficient alternative routes or pathways. Blockage of the cerebral venous drainage through the jugular foramen can cause raised intracranial pressure, which can lead to epileptic seizures, however, in our case the presentation was of unilateral palsy of lower CNs. 

Multiple CN palsies are often a diagnostic challenge because the nerves can be affected at any site along their course. In our case, CN IX–XII were involved which suggested a pathological process located near the jugular foramen. This foramen courses anterolaterally as it exits the skull base and consists of a smaller anteromedial portion (the pars nervosa) and a larger posterolateral portion (the pars vascularis) that are separated by a complete or incomplete fibrous or bony septum. The pars nervosa contains CN IX–XI, the inferior petrosal sinus, and the meningeal branch of the ascending pharyngeal artery. The pars vascularis contains the sigmoid-jugular complex. The jugular foramen varies greatly in size, but averages 15 mm in length and 10 mm in width [10]. In two thirds of cases, the right jugular foramen is larger than the left [10] and is presumed to have some relationship with the dominance of right cerebral sinus drainage. It is interesting to note in this case that CN XI was initially spared.

Involvement of the jugular foramen region leading to lower CN involvement is characterised by several eponymous syndromes, where because of their close anatomical relationship multiple CN involvement is the rule (Table 1). 

Vernet's syndrome is characterised by a unilateral paralysis of CN IX–XI and is due to a lesion inside the skull [11]. CSS refers to dysfunction of CN IX–XII and when this is accompanied by an ipsilateral Horner's syndrome it is termed Villaret's syndrome. CSS is due to extracranial causes but intracranial pathology in theory could also occur [12, 13]. It usually suggests a posterior lacerocondylar space lesion, where these nerves are closely related. CSS appears to be the most likely syndrome related to this presentation despite the initial sparing of the XIth CN. 

Frederic Collet and Jean Sicard provided the original descriptions of CSS based on posttraumatic cases during World War I in which the location of the lesions was demonstrated via radiographic studies as a result of the presence of metallic fragments [12, 13]. Tumours of the ear are the most common cause of CSS [14], but parotid tumours, tumours of the skull base, metastases such as prostate, kidney, breast, melanoma, multiple myeloma and schwannomas of the hypoglossal nerve are also reported [15]. Carcinomatous adenopathies and reticulosis such as Hodgkin's disease have also been reported [14]. Other causes include vascular lesions such as carotid aneurysms [16], jugular vein phlebitis, and especially glomus jugulare tumours [14]. Iatrogenic causes include IJV catheterisation, cerebral angiography, heart surgery, and cerebral vessel clamping [16] as well as surgical emphysema secondary to cryotherapy [17]. Other rarities reported include Lyme disease, polyarteritis nodosa, haemangiopericytoma, idiopathic cranial polyneuropathy [15], and Trousseaus syndrome (migratory thrombophbitis associated with gastric cancer) [1]. Spontaneous thrombophlebitis may be the first manifestation of occult malignancy. In patients with a known malignancy developing features consistent with jugular foramen syndromes, this should always raise the possibility of skull-base metastatic disease. The current literature has only three reported cases of CSS as a result of venous thrombosis [18–20]. This case is interesting, as initially it did not fit into one of the jugular foramen syndromes as CN XI was initially spared. A possible reason for this has already been hypothesised; this is due to the duality of vascularisation between the internal and external branches of CN XI or due to the duality of innervation of these muscles ensured by the external branch of CN XI and C2-4 cervical spinal branches [16].

In this case the cause of thrombosis of the IJV remains unknown, not surprising as 20%–25% of cerebral venous thrombosis have no known aetiology [21]. Despite the preceding symptoms suggesting an infective cause, the patient remained apyrexial with normal inflammatory markers and white cell count throughout. Imaging revealed no infective or neoplastic focii. As preceding infection could not be completely excluded as a cause, antibiotic treatment was commenced in order to prevent septic complications. The thrombophilia, connective tissue, and vasculitic screens were negative, leading us to conclude that the cause of IJV thrombosis in this case appears to be idiopathic. This is the *3rd reported* case of CSS associated with IJV thrombosis, but is certainly the first case where the accessory nerve was initially spared.

When investigating the cause of lower CN palsies, consideration should be given to the previously discussed causes, that is, history of trauma, malignancy, or thrombophilia. Complete examination should be performed including testicular and breast examination as appropriate. Tumour markers may also assist in directing further investigation. A thrombophilia screen should be undertaken in cases where thrombosis is suspected and evidence of connective tissue and vasculitic disease should be sought. Other investigations include audiometry, vestibular testing and if necessary electrophysiological testing of the CNs. 

In terms of imaging, the most sensitive examination is MRI in combination with magnetic resonance venography [22]. T1 weighted and T2 weighted MRI will show a hyperintense signal from the thrombosis. The characteristics of the signal depend on the age of the thrombus and are isointense on T1 weighted images during the first five days and after one month [22]. CT scanning is useful to rule out other acute cerebral disorders and to show venous infarcts or hemorrhages, but it can also be entirely normal. If the diagnosis is still uncertain after MRI or CT venography, conventional cerebral angiography may be indicated.

The poor level of evidence available confounds the management of internal jugular and cerebral sinus thrombosis. Most of the literature relates to solitary case reports or small case series, with no controlled studies due to their rarity. As a result, there is no universal agreement or policy on their management. Certainly a multidisciplinary approach appears to be the key.

The traditional therapy for DVT is anticoagulation with heparin followed by oral anticoagulation. This helps arrest the thrombotic process and to prevent thromboembolic events. Concerns over venous infarction, embolisation, and persistent septic thrombophlebitis have led to recommendations for anticoagulation in patients with sigmoid sinus thrombosis. Embolism has been reported in several studies with an incidence of 0%–33% with the lung being the most frequently affected site [23]. The rate of embolisation has markedly decreased with the introduction and improvement in antibiotics [23]. Bradley et al. suggest that patients with thrombosis confined to the sigmoid sinus should not be anticoagulated to avoid the associated risks; they emphasise that serial imaging with MRI, MR venography, or CT venography is important in all patients to monitor thrombus progression [23]. Criteria supporting anticoagulation include evidence of thrombus progression, extension to other sites on initial examination such as the proximal IJV, transverse sinus or cavernous sinus, neurological changes, persistent fevers or embolic events. Most studies and review articles examining upper extremity DVT recommend anticoagulation therapy [24]. This recommendation is largely based on the concern of the possibility of fatal PE. The other, perhaps better, recommended indication for anticoagulation therapy is to reduce the incidence of postthrombotic syndrome [24]. 

Fibrinolytics are used less and less with surgery such as thrombectomy or partial resection of the vessel being rare nowadays unless it is associated with the surgical treatment of neoplasms [1]. After the acute period, oral anticoagulation with warfarin is typically used for 1–3 months, with a desired INR target of 1.8–2.8. Then the patient is reevaluated with CT or MRI and venography to determine if flow has been reestablished. Prolonged anticoagulant therapy may be required for refractory cases or patients with an underlying prothrombotic state [25].

The optimal management of any venous thrombosis must be individualised and therefore depends on a patient's spectrum of concomitant illness, anticoagulation, bleeding risk, and cardiopulmonary stability. Recent surgery, trauma, active bleeding and thrombocytopenia can impart an increased bleeding risk [26]. Sheikh et al. support the use of low-molecular weight heparin in the acute setting to prevent central propagation, symptomatic pulmonary embolism and maintain any restored venous patency [26]. Superior vena caval filters may be appropriate in patients with IJV thrombosis who are unable to receive anticoagulation and whose cardiopulmonary status is deemed unlikely to tolerate pulmonary embolism of any size [27].

Endovascular thrombolysis can be attempted with the administration of a thrombolytic enzyme, usually urokinase. Published reports are limited to case reports and uncontrolled studies, from which it is impossible to conclude that the results associated with endovascular thrombolysis are superior to those with systemic heparin [28]. Until better evidence is available, endovascular thrombolysis may be applied at centres where the staff have experience in interventional radiology, and this treatment method should be restricted to patients with a poor prognosis. A future randomized trial may compare the effect of heparin with that of endovascular thrombolysis in high-risk patients.

## 4. Conclusion

Due to the complex nature of presentation, delay in diagnosis of CSS is not uncommon. In all cases of suspected CSS, a focal primary lesion or metastatic disease to the temporal bone must be excluded, as early recognition and appropriate treatment may result in significant clinical improvement. Although IJV thrombosis is extremely rare, it should be considered in the differential diagnosis. A multidisciplinary approach to the management of the CSS patient is paramount in achieving successful functional recovery. This case highlights the importance of serial clinical and radiological examinations in assessment of clinical improvement and disease progression in patients with confirmed or suspected CSS.

## Figures and Tables

**Table 1:** Jugular Foramen and related syndromes.

Syndrome	Neurological involvement
Vernet's Syndrome	CN IX, X, XI
Collet-Sicard Syndrome (posterior lacerocondylar space)	CN IX, X, XI, XII
Villaret's Syndrome (Retroparotid space)	CN IX, X, XI, XII, sympathetic chain
Avellis's Syndrome (Brain Stem)	CN X, spinothalamic tract
Schmidt's Syndrome	CN X, spinal XI
Tapia's Syndrome	CN X, XII
Jackson's Syndrome	CN X, XI, XII
